# DAMP signaling in fungal infections and diseases

**DOI:** 10.3389/fimmu.2012.00286

**Published:** 2012-09-10

**Authors:** Cristina Cunha, Agostinho Carvalho, Antonella Esposito, Francesco Bistoni, Luigina Romani

**Affiliations:** ^1^Department of Experimental Medicine and Biochemical Sciences, University of PerugiaPerugia, Italy; ^2^Life and Health Sciences Research Institute (ICVS), School of Health Sciences, University of MinhoBraga, Portugal; ^3^Life and Health Sciences Research Institute/3B’s – Portuguese Government Associate LaboratoryBraga/Guimarães, Portugal; ^4^San Giovanni Battista HospitalFoligno, Italy

**Keywords:** DAMPs, PAMPs, fungal diseases, inflammation, immunoregulation

## Abstract

Fungal infections and diseases predominantly affect patients with deregulated immunity. Compelling experimental and clinical evidence indicate that severe fungal diseases belong to the spectrum of fungus-related inflammatory diseases. Some degree of inflammation is required for protection during the transitional response occurring temporally between the rapid innate and slower adaptive response. However, progressive inflammation worsens disease and ultimately prevents pathogen eradication. The challenge now is to elucidate cellular and molecular pathways distinguishing protective vs. pathogenic inflammation to fungi. In addition to fungal ligands of pattern recognition receptors (pathogen-associated molecular patterns, PAMPs), several host-encoded proteins, the damage-associated molecular patterns (DAMPs), are released during tissue injury and activate innate recognition receptors. DAMPs have been shown to regulate inflammation in fungal diseases. The DAMP/receptor for advanced glycation end-products axis integrated with the PAMP/Toll-like receptors axis in the generation of the inflammatory response in experimental and clinical fungal pneumonia. These emerging themes better accommodate fungal pathogenesis in the face of high-level inflammation seen in several clinical settings and point to DAMP targeting as a novel immunomodulatory strategy in fungal diseases.

## INTRODUCTION

Fungi are associated with a wide spectrum of diseases in humans and animals, ranging from acute self-limiting pulmonary manifestations and cutaneous lesions in immunocompetent individuals to severe inflammatory diseases and life-threatening invasive infections in immunocompromised patients. Most fungi are ubiquitous in the environment and humans are exposed by inhaling spores. The ability to colonize almost every niche within the human body involves specific reprogramming events to adapt to environmental conditions (Cooney and Klein, 2008). In the case of commensals, such as *Malassezia* spp. and *Candida albicans*, co-evolution with their mammalian hosts for millions of years implicates the existence of complex mechanisms of immune adaptations and, likewise, of sophisticated mechanisms to antagonize immunity.

Indeed, most fungi are considered harmless in the context of normal host responses indicating that a stable pathogen–host interaction is common for microorganisms with inherently low virulence. This implicates that a high degree of coexistence occurs between fungi and their mammalian hosts, which deviates into overt disease only under specific conditions, most prominently deficits in resistance and tolerance mechanisms, respectively, defined as the ability to limit pathogen burden or the damage caused by the infection and/or the host (Romani, 2011).

## INFLAMMATION: THE GOOD, THE BAD, AND THE UGLY

As in autoimmunity and chronic inflammation, an imbalance between pro- and anti-inflammatory signals may prevent successful host/fungal interaction, thus leading to infection and disease (Romani and Puccetti, 2007). Indeed, despite the occurrence of severe fungal infections in immunocompromised patients, clinical evidence indicate that fungal diseases also occur in the setting of an heightened inflammatory response, in which immunity occurs at the expense of host damage and pathogen eradication (Perfect, 2012). It is known that aberrant stimulation of Toll-like receptors (TLRs) by damage-associated molecular patterns (DAMPs) may result in increased expression of cytokines, chemokines, and proteases, perpetuating a vicious inflammatory cycle that constitutes the hallmark chronic inflammatory human diseases (Srikrishna and Freeze, 2009; Piccinini and Midwood, 2010). We have recently described an additional mechanism by which host inflammation may favor fungal infectivity and promotes the transition from fungal commensalism to infection. Fungal sensing of the mammalian cytokine interleukin (IL)-17A induced artificial nutrient starvation conditions in *C. albicans* and *Aspergillus fumigatus*, two major human fungal pathogens, resulting in increased adhesion and filamentous growth that clinically translates in a dramatic increment of biofilm formation and fungal virulence (Zelante et al., 2012). Thus, commensals or ubiquitous fungi have evolved a contingency-based system during co-evolution to guarantee their persistence in an inflammatory host environment. The main implication of these findings is that, at least in specific clinical settings, it is a heightened inflammatory response that likely compromises a patient’s ability to eradicate infection, and not an “intrinsic” susceptibility to infection that determines a state of chronic or intractable diseases (Romani and Puccetti, 2007). The conceptual principle highlighting a truly bipolar nature of the inflammatory process in infection is best exemplified by the occurrence of severe fungal infections in patients with immune reconstitution syndrome, an entity characterized by localized and systemic inflammatory reactions, worsening disease in opportunistic and non-opportunistic infections, that are associated with immunological recovery (Gupta and Singh, 2011; Perfect, 2012). Additionally, a high incidence of fungal infections and sensitization to *Aspergillus* spp. has been described in the hyper-IgE syndrome in which increased levels of pro-inflammatory gene transcripts have been found (Antachopoulos et al., 2007; Holland et al., 2007). Therefore, paradoxically, increased inflammatory innate response may predispose to either fungal infections or deregulated immune responses to the fungus. Thus, fungal diseases represent an important paradigm in immunology since they can result from either the lack of recognition or over-activation of the inflammatory response.

## INNATE RECOGNITION OF FUNGI

### VIA PATHOGEN-ASSOCIATED MOLECULAR PATTERNS

**FIGURE 1:**
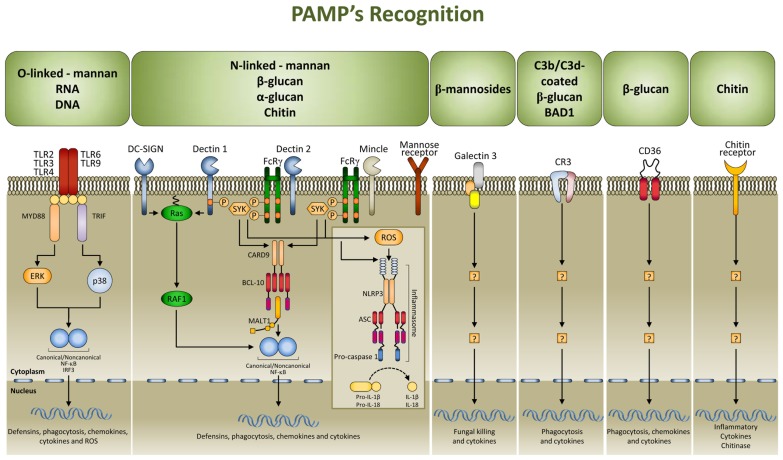
**Signaling pathways in fungal pathogen-associated molecular patterns (PAMPs) recognition.** PAMPs are recognized by pattern recognition receptors (PRRs). The major PRRs are Toll-like receptors (TLRs); C-type lectin receptors [CLRs; such as dectin 1, dectin 2, DC-specific ICAM3 grabbing non-integrin (DC-SIGN), mincle, and the mannose receptor], galectin family proteins (such as galectin 3), and the class B scavenger receptor CD36. TLRs and CLRs activate multiple intracellular pathways upon binding to specific fungal PAMPs, including β glucans [especially β (1,3)-glucans with varying numbers of β (1,6) branches], chitin, a polymer of *N*-acetylglucosamine, mannans linked to proteins through N or O linkages, β (1,2) linked oligomannosides, and fungal nucleic acids. These signals activate canonical or non-canonical nuclear factor-κB (NF-κB) and the NOD, LRR- and pyrin domain-containing 3 (NLRP3) inflammasome, and this culminates in the production of defensins, chemokines, cytokines, and reactive oxygen species (ROS). CR3, complement receptor 3; ASC, apoptosis-associated speck-like protein containing a CARD; BCL 10, B cell lymphoma 10; CARD9, caspase recruitment domain-containing protein 9; ERK, extracellular signal-regulated kinase; FcRγ, Fc receptor γ chain; IL, interleukin; IRF3, IFN-regulatory factor 3; MALT1, mucosa-associated lymphoid tissue lymphoma translocation protein 1; MYD88, myeloid differentiation primary response protein 88; SYK, spleen tyrosine kinase.

Multiple cell populations and cell-signaling pathways are involved in the antigen-independent recognition of the fungus by the innate immune system. Applying systems biology approaches to this complex process has resulted in a better appreciation of the intricate cross-talk provided by temporal changes in mediators, metabolites, and cell phenotypes underlining the coordinated processes (Santamaria et al., 2011). Pattern recognition receptors (PRRs) for fungal pathogen-associated molecular patterns (PAMPs) include TLRs, C-type lectin receptors, nucleotide oligomerization domain-like receptors (NLRs), and NALP3 inflammasome (Romani, 2011; **Figure 1**). Both murine and human studies have confirmed the association of susceptibility to fungal infections and diseases with genetic deficiency of selected PRRs (Cunha et al., 2011a). By varying the composition with the morphotype, growth stage, environment sensing, and fungal species, the cell wall provides the prime sources of PAMPs that, as such, are ideal targets for recognition as non-self by mammalian cells. To achieve optimal activation of antigen-specific adaptive immunity, it is first necessary to activate the pathogen-detection mechanisms of the innate immune response. However, by hyper-inducing pro-inflammatory cytokines, facilitating tissue damage, or impairing protective immunity, PRR activation itself is a double-edged sword and this may explain why PMNs, although essential in initiation and execution of the acute inflammatory response and subsequent resolution, may act as double-edged swords, as the excessive release of oxidants and proteases may be responsible for injury to organs and fungal sepsis (Romani and Puccetti, 2007).

### VIA A DUAL SENSOR SYSTEM

**FIGURE 2:**
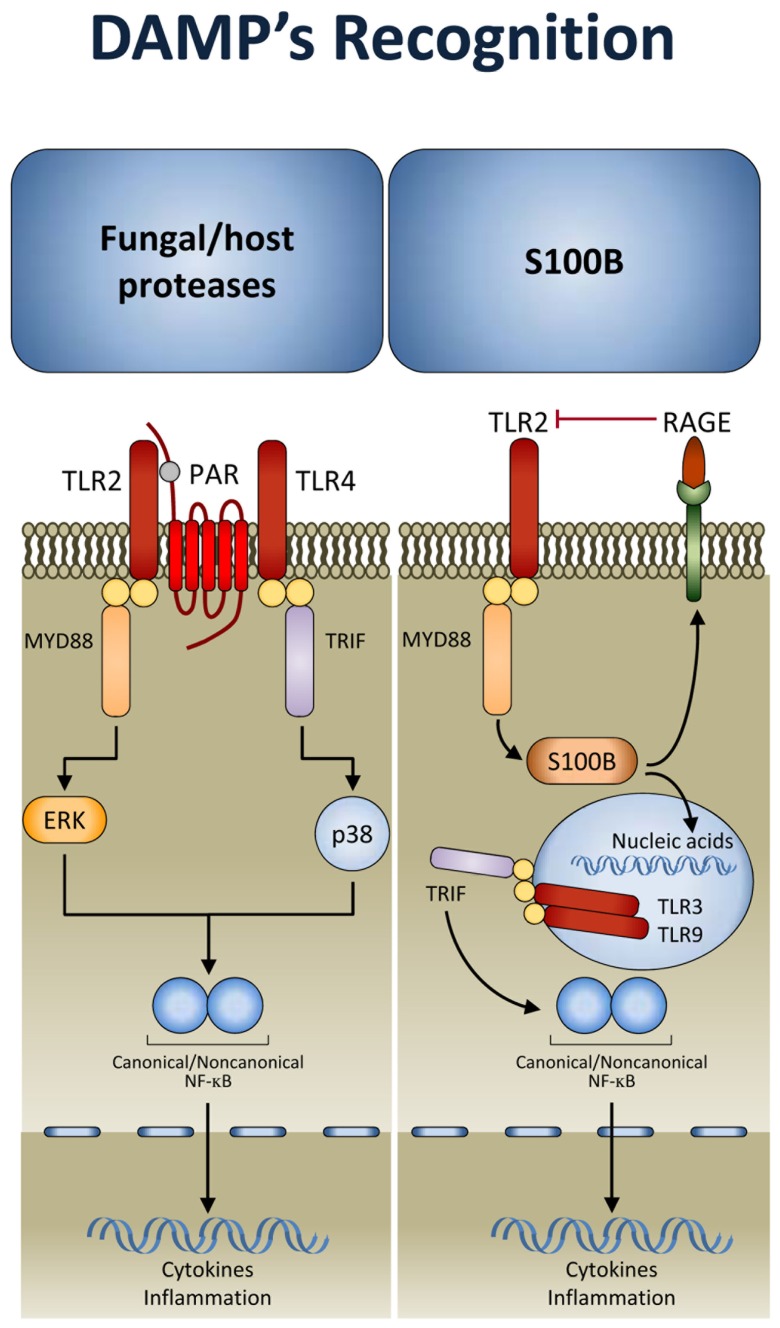
**Damage-associated molecular patterns (DAMP) signaling in response to fungi.** After TLR activation, protease-activated receptors (PARs) sense proteolytic virulence factors and tissue injury and contribute to fungal recognition through a dual sensor system. In addition, the DAMP, S100B, through the spatio-temporal integration of signals from TLRs and RAGE, allows the immune system to discriminate between pathogen-derived and endogenous danger signals (see text for details). TRIF, TIR domain-containing adaptor protein inducing IFN-β; MYD88, myeloid differentiation primary response protein 88; RAGE, receptor for advanced glycation end-products.

There is a growing suspicion that there are additional types of innate immune sensing mechanisms that are not based on pattern recognition but rather on other principles. It is likely that the kind of principles involved are similar to the “guard theory” described in the plant immunity field by Dangl and Jones (2001) whereby the immune system senses the consequences of some stereotypic function of a pathogen or virulence factor. That is, instead of directly sensing the microbial structure, these sensors detect unusual suspicious activities associated with the microbial virulence apparatus. This kind of sensing has been proven to complement pattern recognition mechanisms in fungal infections. Indeed, during inflammation, host- and fungal-derived proteases trigger the activation of protease-activated receptors (PARs), a family of G-protein-coupled receptors (Shpacovitch et al., 2007). It has been shown that activation of TLRs by fungi unmasks an essential and divergent role for PAR1 and PAR2 in downstream signaling and inflammation (**Figure 2**). TLRs activated PARs and triggered distinct signal transduction pathways involved in inflammation and immunity to *C. albicans *and *A. fumigatus*. Inflammation was promoted by PAR1 activation in response to *Candida* and by PAR2 inhibition in response to *Aspergillus*. This occurred by TLR regulation of PAR signaling. Thus, after recognition by TLRs, PARs may become activated to sense proteolytic virulence factors and tissue injury, to mediate inflammatory responses and modulate the activity of TLRs (Moretti et al., 2008). This implicates that fungal recognition by TLRs may be licensed by DAMP recognition (further discussed below). Conceptually, the model is consistent with a binary signaling pathway of mammalian recognition of fungi as observed in *Drosophila*. A fungal protease used by the entomopathogenic fungus *Beauveria bassiana* to digest the cuticle was shown to activate the Toll pathway by inducing the maturation of Persephone into an active protease (Gottar et al., 2006). Thus, many of the mechanisms used by the innate immune system in animals show surprising parallels with those of immunity in plants. Ultimately, sensing pathogen and virulence is a dual sensor system to detect fungi that works throughout evolution.

### VIA DAMAGE-ASSOCIATED MOLECULAR PATTERNS

Inflammation results from recognition of PAMPs and DAMPs (Gallucci and Matzinger, 2001). Despite the identification of specific signaling pathways negatively regulating responses to PAMPs or DAMPs (Bianchi, 2007), the unexpected convergence of molecular pathways responsible for recognition of PAMPs and DAMPs raised the question of whether and how the host discriminates between PAMPs and DAMPs and the relative contribution of either one to inflammation, immune homeostasis, and mechanisms of repair during infection. It seems clear that the strategy of innate immunity to identify and specifically respond to the presence of a broad class of pathogens and injuries is based on the existence of a set of PRRs that can discriminate between PAMPs and DAMPs. The combined activation of these different receptors by their specific ligands triggers signaling pathways that can be complementary, synergistic, or antagonistic (Lee and Kim, 2007; Trinchieri and Sher, 2007). DAMPs such as the high mobility group box 1 protein and S100 proteins represent important danger signals that, although primarily intracellular, may mediate inflammatory responses through autocrine/paracrine interactions with the receptor for advanced glycation end-products (RAGE), a multiligand receptor of the immunoglobulin superfamily (Schmidt et al., 2001; Donato, 2007; Sparvero et al., 2009). Integral to the biology of RAGE and its ligands is their up-regulation and increased accumulation in multiple biological and disease settings. The ability to activate expression programs that encode innate immune responsive genes confers to RAGE a central role in chronic inflammatory diseases. Engagement of RAGE converts a brief pulse of cellular activation to sustained cellular dysfunction, eventually leading to inflammation and tumor promotion. However, because RAGE is expressed in multiple, distinct cell types, including immune cells, and both murine and human RAGE gene undergoes extensive splicing with distinct splice isoforms being uniquely distributed in different tissues (Kalea et al., 2009), it is not surprising that diverse signal transduction and effector pathways may be impacted by RAGE depending on sites, ligands and time course of ligand–RAGE stimulation (Donato, 2007).

A mechanism that discriminates between fungi- and danger-induced immune responses via the spatio-temporal integration of signals from TLRs and RAGE has recently been described (**Figure 2**). The mechanism exploits a previously unrecognized role for the S100B/RAGE axis that, in sensing danger, plays a critical and unanticipated role as a fine modulator of inflammation in experimental (Sorci et al., 2011) and human (Cunha et al., 2011b) fungal pneumonia. By forming complexes with various TLR ligands, S100B exhibited promiscuous activities at the extracellular and intracellular levels. It inhibited TLR2 via RAGE, through a paracrine epithelial cells/neutrophil braking circuit, and this accounted for its anti-inflammatory activity in infection. However, the ability of S100B to bind nucleic acids resulted in the activation of intracellular TLRs converging on TRIF and eventually resolving danger-induced inflammation via transcriptional down-regulation of S100B gene expression. Thus, in addition to the notion that danger signals may terminate overactive immune responses (Sitkovsky and Ohta, 2005), our study reveals that a pathogen-induced signal may also terminate unnecessary danger-induced injury. A genetically determined hyper-function of the DAMP signaling was indeed associated with invasive aspergillosis in hematopoietic stem cell transplanted patients (Cunha et al., 2011b) and with symptomatic *Candida* vaginitis (Yano et al., 2012). Conceptually, our findings raise the intriguing possibility that the host may have developed mechanisms to ameliorate the response to PAMPs via DAMPs. This is also exemplified by signaling through TLR3. In addition to activation by viral double-stranded RNA, TLR3 can be activated by endogenous mRNA released by necrotic cells (Kariko et al., 2004), a mechanism by which epithelial injury may lead to inflammation. TLR3 plays a non-redundant role in the induction of immunological tolerance in fungal infections (De Luca et al., 2007,^2010^), a finding highlighting the contribution of sensing danger in response to fungi. On a translational level, targeting DAMP signaling may be of therapeutic benefit in high-risk patients. We have recently found that a hyper-function of the DAMP signaling underlies inflammation in response to airborne fungi in mice with cystic fibrosis and obtained a proof-of-concept demonstration that the inhibition of RAGE could be of therapeutic benefit in these mice (unpublished observations). Together, these findings confirm that an injudicious response to PAMPs may result in a hostile environment to beneficial commensal fungi. Therefore, discrimination between pathogenic forms, causing cellular damage and tissue injury, and innocuous commensals may be critical in maintaining the balance and tissue homeostasis.

## DANGER SENSING IN RESPONSE TO FUNGI: THE PIVOTAL ROLE OF INFLAMMASOMES

Among PRRs, the cytosolic NLR family member NLRP3 (also known as NALP3, cryopyrin, and CIAS1) is a key player in host defense against *C. albicans* (Gross et al., 2009; Hise et al., 2009; Joly et al., 2009; Joly and Sutterwala, 2010). A role for NALP3 has also been shown in response to *A. fumigatus* (Said-Sadier et al., 2010). NLRs are a family of intracellular proteins with a tripartite modular structure that contain a central nucleotide-binding oligomerization domain, a C-terminal LRR, and an N-terminal effector-binding domain that shares structural similarity with a subclass of plant disease resistance genes. Several NLRs (NALPs and IPAF subfamilies) form multi-protein complexes termed inflammasomes which activate inflammatory pro-caspases and the subsequent processing and secretion of IL-1β, IL-18, and IL-33 (**Figure 1**). As intracellular receptors, the NLR family is in a prime location to detect danger signals associated with host stress and may therefore play a critical role in recognizing the transition from commensal to pathogen (Joly and Sutterwala, 2010). The activation of the NLRP3 inflammasome result in the activation of caspase-1 and processing and secretion of IL-1β that mediates strong innate antifungal responses and regulates Th1/Th17 cell activation (Bellocchio et al., 2004; Vonk et al., 2006; Bozza et al., 2008; Hise et al., 2009; van de Veerdonk et al., 2011). As a matter of fact, mice deficient for IL-1R (the receptor for IL-1β) are resistant and mice with hyper-functioning of the IL-1β signaling are susceptible to candidiasis. In addition, polymorphisms in the gene coding for NLRP3 have been associated with recurrent vulvovaginal candidiasis (Lev-Sagie et al., 2009), a finding consistent with the notion that deregulated NLRP3 inflammasome activation is associated with both heritable and acquired inflammatory diseases.

Currently, three models exist to explain NLRP3 inflammasome activation: (a) lysosomal disintegration and release of its content by phagocytosed material; (b) induction of reactive oxygen species production at mitochondrial membranes; and (c) potassium efflux by membrane channels or ionophoric compounds (Mankan et al., 2012). While it is accepted that *C. albicans* live yeasts induce caspase-1-mediated IL-1β secretion in a NLRP3-dependent manner (Gross et al., 2009; Hise et al., 2009; Joly et al., 2009; Kumar et al., 2009), the precise pathways involved in NLRP3 inflammasome activation are yet to be defined. Although hyphae did not possess stimulatory properties, the ability of *C. albicans* to transition from yeast to hyphal form was essential for NLRP3 activation (Joly et al., 2009). Thus, irrespective of the nature of the ligands that activate NLRP3 in *C. albicans* infection, sensing danger by the NLRP3 inflammasome, and likely NLRC4 (Tomalka et al., 2011), is an inherent component of the host control of fungal opportunism and infectivity.

## CONCLUSION

It is now clear that several DAMPs are vital danger signals that alert the immune system to tissue damage upon fungal infections. However, PRR activation by DAMPs may initiate positive feedback loops where increasing tissue damage perpetuates pro-inflammatory responses leading to chronic inflammation. Indeed, DAMPs have been implicated in fungal diseases where excessive inflammation plays a key role in pathogenesis, including fungal pneumonia in transplanted patients and recurrent vulvovaginal candidiasis. Dissection of the reciprocal regulation between DAMP and PAMP signaling pathways, and the relative contribution of either one, in fungal diseases may increase our understanding of the pathogenesis of these diseases with the ultimate goal of developing new strategies that target DAMPs to selectively modulate the immune response to fungi.

## Conflict of Interest Statement

The authors declare that the research was conducted in the absence of any commercial or financial relationships that could be construed as a potential conflict of interest.
